# Quantification of Nanovoids in Fully-Aromatic Thin-Film Composite Polyamide Membranes Using a Quartz Crystal Microbalance Equipped with a Humidity Cell

**DOI:** 10.1021/acs.est.6c00927

**Published:** 2026-07-25

**Authors:** Chenyue Wu, Zhe Yang, Lu Elfa Peng, Pulak Sarkar, Chuyang Y. Tang

**Affiliations:** † Department of Civil Engineering, 25809 The University of Hong Kong, Pokfulam, Hong Kong SAR, 999077 China; ‡ Department of Civil and Environmental Engineering, The Hong Kong University of Science and Technology, Hong Kong SAR, 999077 China; § Department of Chemical and Biological Engineering, The Hong Kong University of Science and Technology, Hong Kong SAR, 999077 China; ∥ Department of Civil & Environmental Engineering, 37580 National University of Singapore, Singapore 117576, Singapore

**Keywords:** thin-film composite (TFC) membranes, nanovoids, polyamide, quartz crystal microbalance (QCM), humidity cell

## Abstract

The design of thin-film composite (TFC) membranes is crucial for efficient separation, as the transport of water and solutes is governed by the intrinsic microstructure of its polyamide selective layer. The presence of nanovoids in the top polyamide selective layer significantly impacts membrane separation performance and regulates water transport pathways through the film. In this study, we present a novel approach that employs a quartz crystal microbalance (QCM) equipped with a humidity cell to characterize the size and distribution of nanovoids in the polyamide layers of five commercial TFC membranes. By utilizing the Kelvin equation and accurately measuring the mass changes under a controlled humidity environment, we quantified the nanovoids, detecting small voids often overlooked by traditional methods. Our findings offer critical structural parameters, including void size distribution, total void volume, and void fraction, providing a practical and accessible alternative to complex methods. The findings were also validated and compared with traditional microscopic analysis, revealing that the smaller voids are often overlooked by the transmission electron microscopy characterization. Overall, this study demonstrates the feasibility of employing QCM humidity cells to quantify nanovoids and provides a useful tool for membrane characterization, fostering future advancements in membrane design and performance optimization.

## Graphical abstract

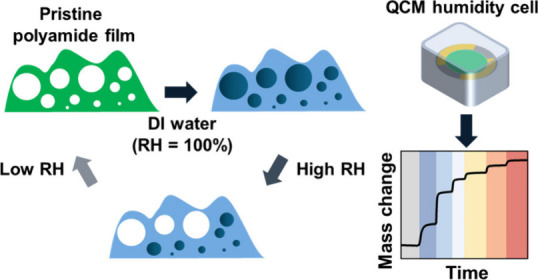

## Introduction

Thin-film composite (TFC) membranes, the leading standard for reverse osmosis (RO) and nanofiltration (NF) technology, play a critical role in various desalination, water treatment, and water reuse processes for augmenting potable water.^1–3^ These membranes consist of three key layers: a thin polyamide selective layer, a middle porous support layer, and a base nonwoven fabric.^1,4–7^ To achieve relatively high NaCl rejection, the polyamide layers of commercial membranes (e.g., SW30HR, XLE, NF90, *etc*.) are commonly formed by the interfacial polymerization of *m*-phenylenediamine (MPD) and trimesoyl chloride (TMC), resulting in a fully aromatic, highly cross-linked polyamide layer.^8–11^ This layer, with a typical apparent thickness of a few hundred nanometers, exhibits a distinctive ″ridge-and-valley″ rough surface morphology harboring many nanovoids in the size range of a few nm to over 100 nm.^8,12–14^ These nanovoids enlarge the effective filtration area,^15–17^ regulate water and solute transport through a self-gutter effect,^16,18^ and thereby significantly impact membrane performance.

Traditional microscopic techniques, notably transmission electron microscopy (TEM), have greatly advanced the understanding of the nanovoid structure within the polyamide selective layer.^19,20^ The cross-sectional TEM analysis has confirmed the presence of a significant number of nanovoids,^21^ with their volume fractions reaching up to approximately 30%.^19,20^ Although this approach provides a reasonable estimation of the void fraction, it tends to underestimate void sizes (Supporting Information S1). More recently, electron tomography (ET) has also been successfully employed to characterize the three-dimensional (3D) nanovoid-containing microstructure of polyamide layers.^8,22–26^ Furthermore, cryo-ET was used to characterize polyamide desalination membranes, showing a total void fraction in the range of 18 to 22% for commercial polyamide membranes such as BW30 and SW30XLE.^27^ Despite its highly effective and detailed structural analysis, the 3D tomography method faces challenges such as high costs, the need for specialized equipment and expertise, complex sample preparation, and limited accessibility. These constraints highlight the need for more affordable and accessible methods to complement the existing characterization techniques for membrane microstructures.

A recent study by Coronell and colleagues^19^ introduced quartz crystal microbalance (QCM) to quantify nanovoid fractions for various commercial RO membranes. Based on water uptake measurements using a QCM flow cell, they reported that nanovoids constitute a significant volume fraction (15–60%) within the polyamide layers.^19^ This method, also adopted by other research groups,^28,29^ offers an elegant and more affordable alternative to microscopy/tomography-based techniques.^19^ Unfortunately, this simple QCM method developed by Coronell and co-workers provides little structural information on the size and distribution of nanovoids.^19^

To overcome the limitation of Coronell’s work, we envisage the application of the QCM technique under a precisely controlled humidity environment. Based on previous studies on water condensation/evaporation in nanopores, the equilibrium water vapor pressure (and thus the relative humidity (RH)) is directly related to the pore size according to the Kelvin equation.^30–33^ In the current study, we report a novel characterization method for accessing nanovoid size and distribution using a QCM humidity cell (Figure 1a and additional details in Supporting Information S2). This dedicated cell comprises a liquid compartment and a vapor compartment, separated by a hydrophobic membrane. By injecting a carefully selected saturated salt solution into the liquid compartment, the RH in the vapor compartment can be systematically controlled (Figure 1b). Thus, by exposing a fully wetted polyamide layer to progressively reduced RH, water evaporation will first take place in larger nanovoids, followed by smaller ones (Figure 1c). Monitoring the mass change during this process using QCM (Figure 1d) allows the determination of nanovoid volumes over specific void size ranges (Figure 1b), resulting in a detailed size distribution of these nanovoids.

**Figure 1:**
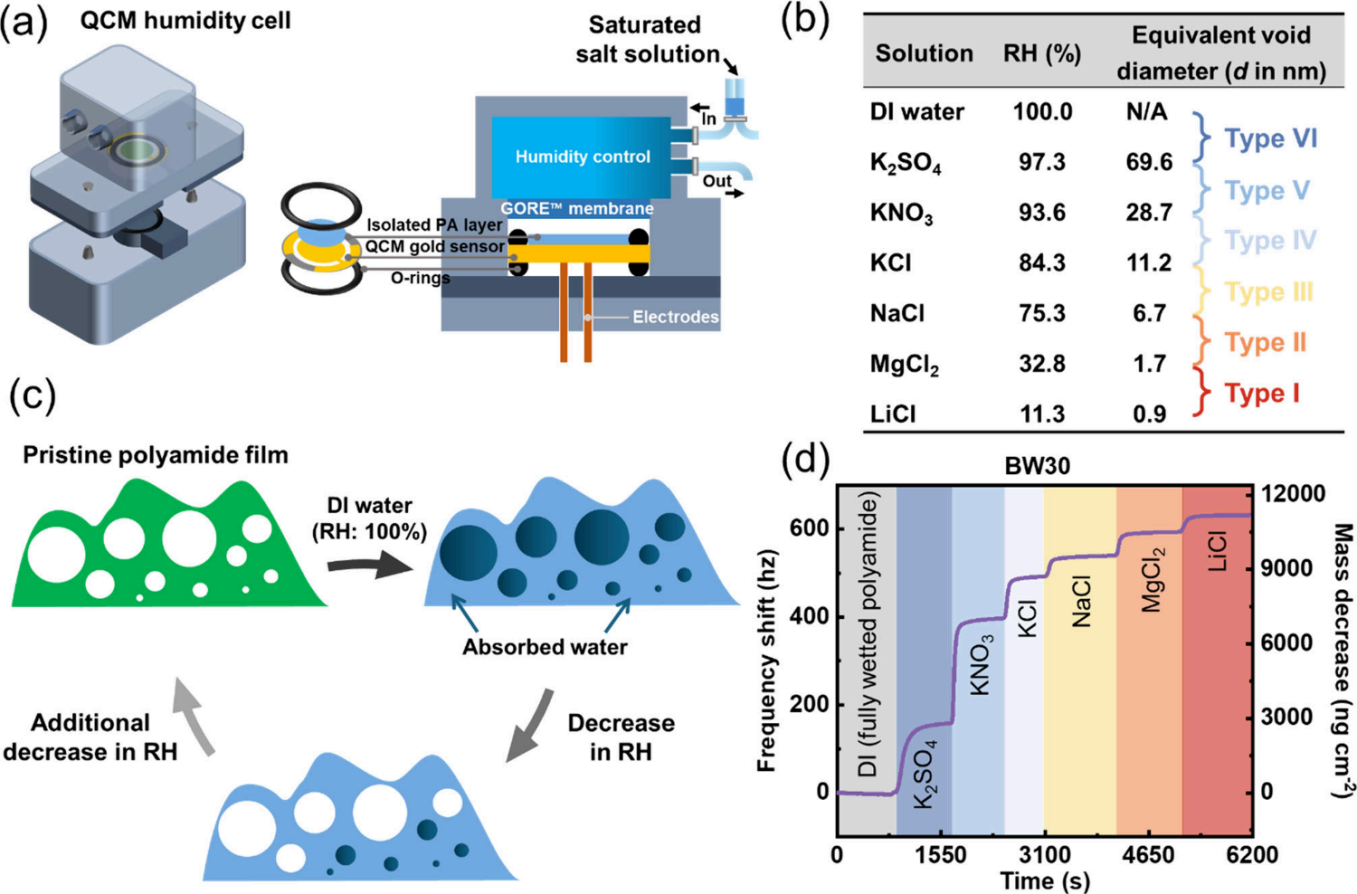
(a) The schematic illustration of the 3D and cross-sectional view of the QCM humidity cell used in this study. The QCM humidity cell includes a liquid compartment of salt solution and a vapor compartment, separated by a GORE membrane. The isolated PA layer is exposed to the vapor compartment, and it is not in direct contact with the salt solution. (b) The details of relative humidity (RH) associated with different saturated salt solutions and corresponding equivalent void diameter calculated using Kelvin equation.^34,35^ Six void size categories were defined based on their ranges: Type I (<1.7 nm), Type II (1.7–6.7 nm), Type III (6.7–11.2 nm), Type IV (11.2–28.7 nm), Type V (28.7–69.6 nm), and Type VI (>69.6 nm). (c) The mechanism governing the absorption and desorption of water molecules from the nanovoids within the polyamide selective layer under varying relative humidity (RH%) during the experiment. (d) The data of the frequency shift and corresponding mass change for the BW30 membrane when various saturated salt solutions were injected into the liquid compartment.

In this study, we demonstrate the feasibility of QCM humidity cell for the systematic quantification of nanovoids for five commercial TFC membranes. Our approach provides key structural parameters such as void size distribution, total void volume, and void fraction in their polyamide layers. The method also enables the detection of small voids that are often overlooked by traditional 2D TEM analysis. This novel technique provides an alternative approach to nanovoid characterization, offering detailed void information to advance the development and optimization of TFC membranes.

## Materials and Methods

### Materials and Chemicals

Five fully aromatic polyamide TFC membranes, including two seawater reverse osmosis membranes (SW30HR and SW30XLE), two brackish water reverse osmosis membranes (BW30 and XLE), and one nanofiltration membrane (NF90), were obtained from DuPont Water Solutions (US). Dimethylformamide (DMF, 98%) was obtained from Sigma-Aldrich (China). Potassium sulfate (K_2_SO_4_), potassium nitrate (KNO_3_), potassium chloride (KCl), sodium chloride (NaCl), magnesium chloride (MgCl_2_), and lithium chloride (LiCl) were used to prepare saturated solutions for humidity control (see details in Table S1 and Supporting Information S3). All aqueous solutions were prepared using deionized (DI) water produced from the Milli-Q system with a resistivity of 18.2 MΩ cm (Millipore, US).

### Microscopic Characterization of Polyamide Selective Layers

The surface morphology of polyamide TFC membranes was examined by using scanning electron microscopy (SEM, Hitachi S4800 FEG SEM, Japan). The surface roughness of the polyamide films was characterized by atomic force microscopy (AFM, Bruker, Germany). TEM (Philips CM100, Japan) was employed to visualize the cross-sectional structure and measure the thickness (i.e., apparent and intrinsic thickness) of the polyamide layers. For TEM analysis, resin-embedded membrane samples were sliced into ultrathin sections (<100 nm) using a Leica EM UC7 (Leica Microsystems, Wetzlar, Germany).^36,37^ The void fraction of the polyamide selective layers was quantified by analyzing TEM images with Image-Pro Plus software (Media Cybernetics, Inc.).^21^

### Humidity Controlled QCM Experiment

A polyamide layer was isolated from a TFC membrane and loaded onto a gold-coated quartz sensor (see details in Supporting Information S4).^38,39^ The Q-Sense E4 system (Biolin Scientific, Sweden) was employed to determine the mass changes of the isolated polyamide layer by monitoring the frequency shift of the sensor, in accordance to the Sauerbrey equation:^40,41^

1 $$\Delta f=-\frac{2f^{2}}{S_{q}\sqrt{\rho_{q}\mu_{q}}}\Delta m$$

where *Δf* is the frequency shift (Hz), *f* is the resonant frequency (Hz) of the quartz sensor, *S*_*q*_ is the active area of the quartz sensor (m^2^), *ρ*_*q*_ is the density of quartz (kg m^–3^), *μ*_*q*_ is the shear modulus of quartz (Pa), and *Δm* is the mass loaded onto the quartz sensor (kg).

The dry mass of the isolated polyamide layer was measured in a Q-Sense flow cell (QFM 401, Biolin Scientific). In addition, to investigate mass changes under controlled humidity conditions, the polyamide-loaded sensor was mounted into a Q-Sense humidity cell (QHM 401, Biolin Scientific, Figure 1a and Figure S2). DI water (RH = 100%) was injected into the liquid compartment of the humidity cell, which allows the nanovoids contained in the polyamide to be completely filled with water. The total water uptake (ng cm^–2^) by the nanovoid-containing polyamide film was measured. The injection liquid was then replaced with a series of saturated salt solutions in a sequential manner (Figure 1b,d), including K_2_SO_4_ (RH = 97.3%), KNO_3_ (RH = 93.6%), KCl (RH = 84.3%), NaCl (RH = 75.3%), MgCl_2_ (RH = 32.8%), and LiCl (RH = 11.3%). For each solution, the water flow was maintained at 80 μL min^–1^ over an equilibrium time of at least 10 min. This systematically programmed reduction in RH removes water from wetted nanovoids, starting from bigger ones, followed by smaller ones (Figure 1c), according to the Kelvin equation:

2 $$\mathrm{In}\frac{P_{v}}{P_{sat}}=-\frac{4\gamma V_{m}\;\cos\left(\theta \right)}{dRT}$$

where *P*_*v*_ is the equilibrium vapor pressure, *P*_*sat*_ is the saturated vapor pressure over a flat water surface, γ is the surface energy of water (0.072284 N m^–1^), *V*_*m*_ is the molar volume of water (1.8 × 10^5^ mol m^–3^), *R* is the gas constant (8.314 J mol^–1^ K^–1^), *T* is the absolute temperature (K), and *d* (m) is the void diameter. In this study, the wetting angle θ is taken as 0°, since the polyamide layer is fully wetted with water at the start of the experiment. Under thermodynamic equilibrium with the surrounding air, the RH (%) can be defined as 100·(*P*_*v*_/*P*_*sat*_). Therefore, for spherically shaped voids in a completely wetted membrane, the void diameter can be determined by (Figure 1b,c):

3 $$d=-\frac{4\gamma V_{m}}{RT\;\ln\left(\mathrm{RH}/100\right)}$$

In reality, nanovoids in polyamide membranes are generally heterogeneous in shape, often elongated or irregular, leading to high aspect ratios that deviate significantly from a spherical model. In this case, a more general form of Kelvin equation is applicable:

4 $$\mathrm{In}\frac{P_{v}}{P_{sat}}=-\frac{\gamma V_{m}\;\cos\left(\theta \right)}{RT}\left(\frac{1}{r_{1}}+\frac{1}{r_{2}}\right)$$

where *r*_1_ and *r*_2_ are the principal radii of curvature of the meniscus along its perpendicular axes, and the expression (1/*r*_1_ + 1/*r*_2_) represents twice the mean curvature. Consequently, *d* obtained from Equation 3 should be interpreted as an equivalent void diameter. For nonspherical voids, this equivalent diameter *d* is related to the principal radii of curvature by

5 $$4/d=\left(1/r_{1}+1/r_{2}\right)$$

For example, for elongated voids with high aspect ratio, *r*_2_ can be much larger than *r*_1_ (with *r*_2_ = ∞ for the extreme case of cylindrical-shaped voids), such that *d* ≅ 4 *r*_1_. Therefore, it is worthwhile to explicitly note that the calculated void size represents an equivalent diameter rather than the true physical diameter of the voids. Despite this limitation, it still provides useful complementary information to conventional electron microscopy-based methods.

By sequentially injecting saturated salt solutions with decreasing RH, water is removed from nanovoids of decreasing size, and the corresponding mass changes and void sizes are derived from Equation 1 and 3 (Figure 1d). For example, injecting saturated K_2_SO_4_ solution into the liquid compartments corresponds to a calculated void size of 69.6 nm, indicating that water will evaporate from voids larger than 69.6 nm. Similarly, other salt solutions yield their respective void sizes (Figure 1b). By this method, switching between two solutions allows us to identify the size range of nanovoids within the membrane. In the current study, the entire range of nanovoid sizes was categorized into six types: Type I (<1.7 nm), Type II (1.7–6.7 nm), Type III (6.7–11.2 nm), Type IV (11.2–28.7 nm), Type V (28.7–69.6 nm), and Type VI (>69.6 nm). Additionally, void volume, void fraction, and their distribution for the different void size ranges were calculated from the corresponding mass changes at different RH levels (see Supporting Information S5 for details). Strictly speaking, the Kelvin equation is not directly applicable to very small voids where molecular interactions can significantly affect water evaporation (particularly for Type I voids). In such cases, the measured change in the mass of water may also include contributions from water absorbed in the polyamide matrix and the surface coating in addition to the water filling the nanovoids (Figure 1c). In the current study, two independent samples were measured for each membrane type.

## Results and Discussion

### Microscopic Characterization of Polyamide Selective Layers

Figure 2a-c presents the micrographs of the top surfaces (SEM and AFM) and cross sections (TEM) of the TFC membranes. These membranes exhibited a characteristic ″ridges and valleys″ pattern, featuring nodules and leaf-like structures, with surface roughness ranging from 49.9 to 71.0 nm (Table S2), consistent with previous studies.^13,21,27^ The cross-sectional TEM images (Figure 2c) show a clear visualization of the crumpled structure of the polyamide layer, revealing the presence of a layer of nanovoids-containing densely packed nodular and leaf-like features.^36,42^ In addition, NF90, a nanofiltration membrane with relatively high water permeance (Table S2),^43^ also shows an additional exterior layer that consists of largely interconnected, flat, and extended polyamide leaves.^21^ These features are likely caused by interfacial degassing during the interfacial polymerization (IP) reaction.^18,27,36^ Figure 2d presents the void volumes of the membranes based on TEM cross-sectional image analysis (see details in Supporting Information S6), which were classified into the six size groups (i.e., Types I to VI in accordance to Figure 1b). Based on the TEM results, the membranes generally exhibit higher void volume and void fractions for Types IV (11.2–28.1 nm), Type V (28.7–69.6 nm), and Type VI (>69.6 nm) voids (Figure 2 and Table S3). In contrast, smaller voids, including Type I, Type II, and Type III, are often not (or barely) observable. The total void fraction ranges from 23.3%–41.5% (Table S3), which aligns reasonably well with reported values from previous TEM studies.^19^

**Figure 2:**
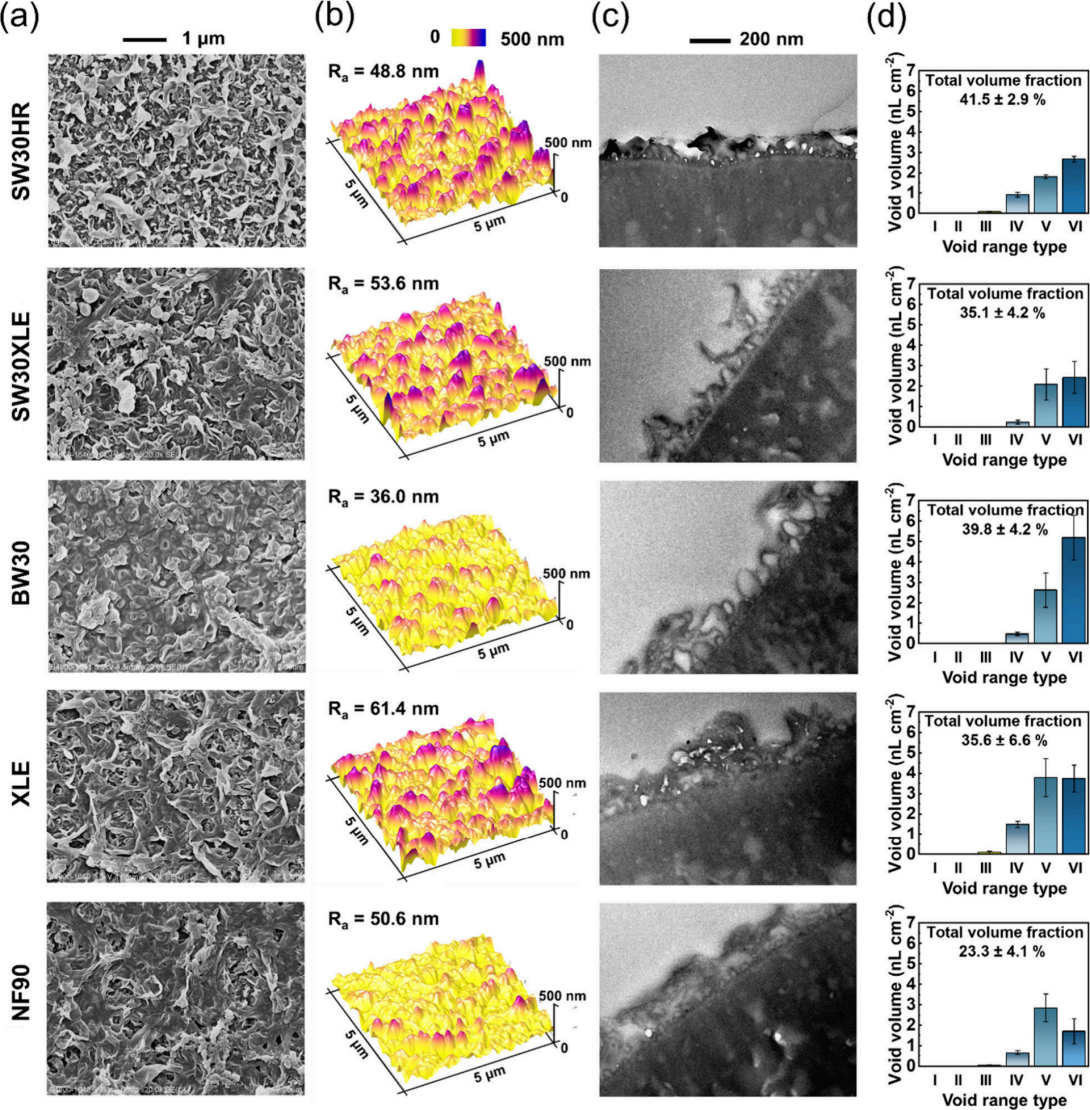
(a) SEM surface morphology, (b) AFM surface morphology, and (c) TEM cross-sectional images of the SW30HR, SW30XLE, BW30, XLE, and NF90 membranes. (d) The results of the void volume in terms of different void ranges of the five commercial TFC membranes that were derived from the TEM cross-sectional images. Image-Pro Plus software was performed to analyze the nanovoid volumes and their distribution in the polyamide layer (further details in Supporting Information S6).^21^ Six void size categories were defined based on their ranges: Type I (<1.7 nm), Type II (1.7–6.7 nm), Type III (6.7–11.2 nm), Type IV (11.2–28.7 nm), Type V (28.7–69.6 nm), and Type VI (>69.6 nm). Two sets of experiments were carried out to duplicate the results for each membrane.

### Characterization of Nanovoids and Their Distribution in Polyamide Selective Layers Using a QCM Humidity Cell

To investigate the distribution of nanovoids and void fraction in the polyamide layer, a QCM equipped with a humidity cell was utilized (Figure 1a). The water desorption experiment for a fully wetted polyamide layer was conducted by monitoring the frequency shift upon sequentially injecting different saturated salt solutions into the liquid compartment (Figure 1b-d). Figure S5 presents the mass of water remaining in the polyamide layers at systematically decreased RH from 100% (DI water) to 11.3% (LiCl). The mass change between two consecutive solutions can be used to quantify the void volumes and their distribution over specific size ranges (Figure 3). Our analysis reveals that all the membranes contain voids of different sizes, ranging from below 0.9 nm (Type I) to over 69.6 nm (Type VI). Overall, the void size distribution aligns reasonably well with the TEM measurements, showing a predominance of larger voids (Types V and VI). However, the QCM method detected a notable presence of smaller voids (Types I to III) that were not easily detected by the TEM method. Nevertheless, NF90 presented an exception: although the TEM results indicate noticeable presence of large voids, the QCM result shows the opposite. This apparent deviation might be attributed to its distinct polyamide morphology, which consists of (1) a nodular layer that contains small nanovoids (Type IV and smaller) and (2) an exterior layer structure composed of large, overlapping leaf-like structures that spread over the membrane surface (see TEM cross-section of NF90 in Figure 2c).^21^ According to Tang and co-workers,^44^ such an exterior layer may result from excessive interfacial degassing during membrane formation. These complicated 3D features, when projected into a 2D plane (such as in the case of 2D TEM), are prone to potential artifacts. For example, two physically disconnected leaf-like features may be mistakenly identified as a single larger one, resulting in wrong identification of “false voids” in TEM characterization (as illustrated in Figure S7). Overall, these comprehensive results shed light on the diverse void characteristics and void volumes of fully aromatic TFC membranes, offering valuable insights into their structural properties.

**Figure 3:**
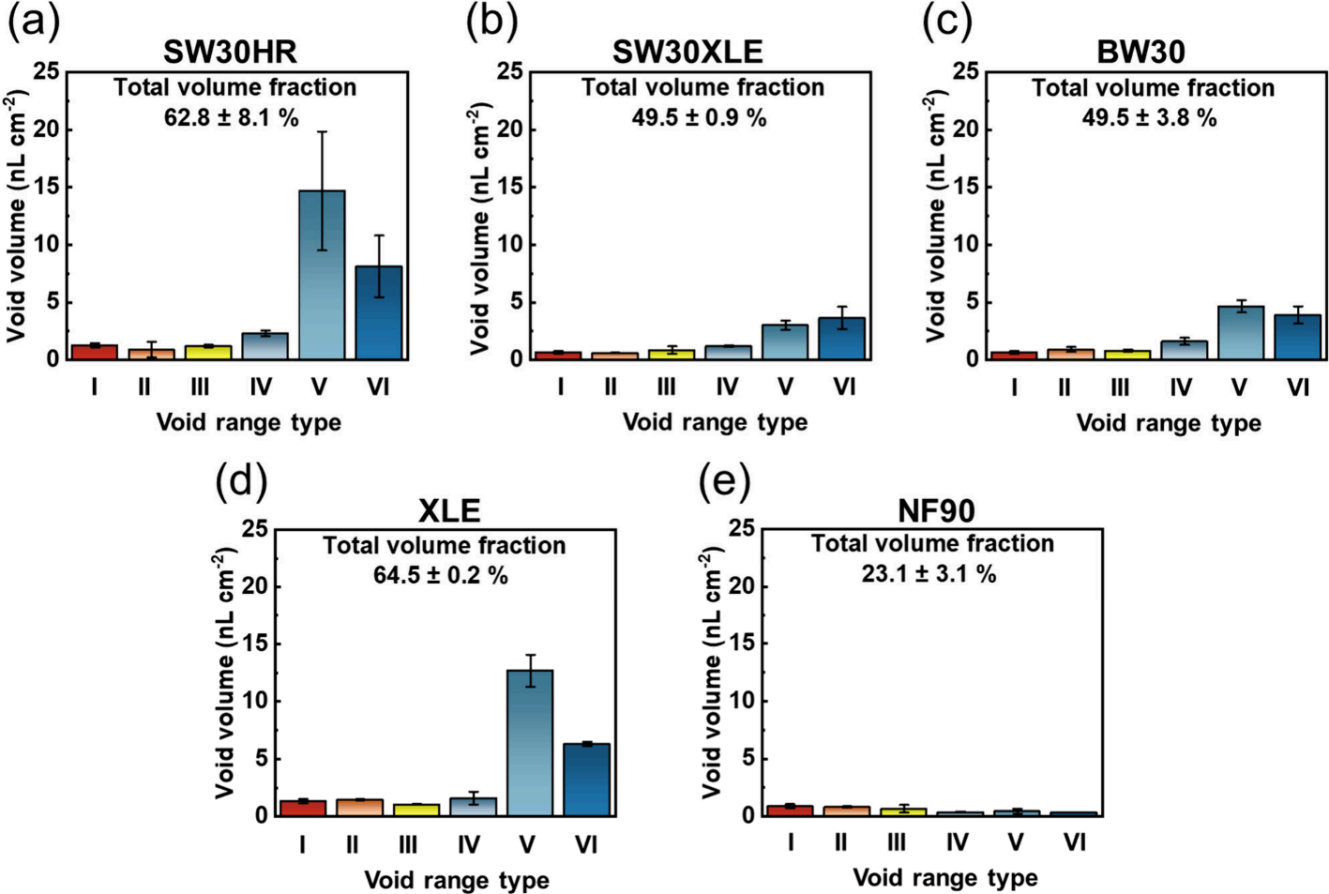
QCM-measured void volume and their distributions for the (a) SW30HR, (b) SW30XLE, (c) BW30, (d) XLE, and (e) NF90 membranes, respectively. Six void size categories were defined based on their ranges: Type I (<1.7 nm), Type II (1.7–6.7 nm), Type III (6.7–11.2 nm), Type IV (11.2–28.7 nm), Type V (28.7–69.6 nm), and Type VI (>69.6 nm). Two sets of experiments were carried out to duplicate the results for each membrane.

Figure 4a presents a comparison of the total void fractions and their distributions across different void sizes of the TFC membranes, as determined by both QCM and TEM methods. The results reveal that void fractions derived from QCM are generally higher than those obtained from TEM measurements. In addition, the total void fractions measured in this study are generally comparable with the literature data reported by Lin et al.^19^ (Figure 4b). It is observed that the void fractions measured by QCM for larger voids (Types IV to VI) align well with the TEM-measured void fractions. While both QCM and TEM are capable of detecting larger voids, the smaller voids (Types I to III) are often missed or overlooked in TEM analysis (Figure 4a and Table S2). As a result, TEM measurement tends to underestimate the calculation of the total void fractions. This discrepancy could be attributed to the inherent limitations associated with TEM sample preparation and testing, such as resin embedding or vacuum processes. In addition, its application is constrained by sample area, insufficient contrast between the polyamide and background, subjective measurements, and challenges with pixel resolution, which can impact the accuracy and reliability of interpretations.^27^

**Figure 4:**
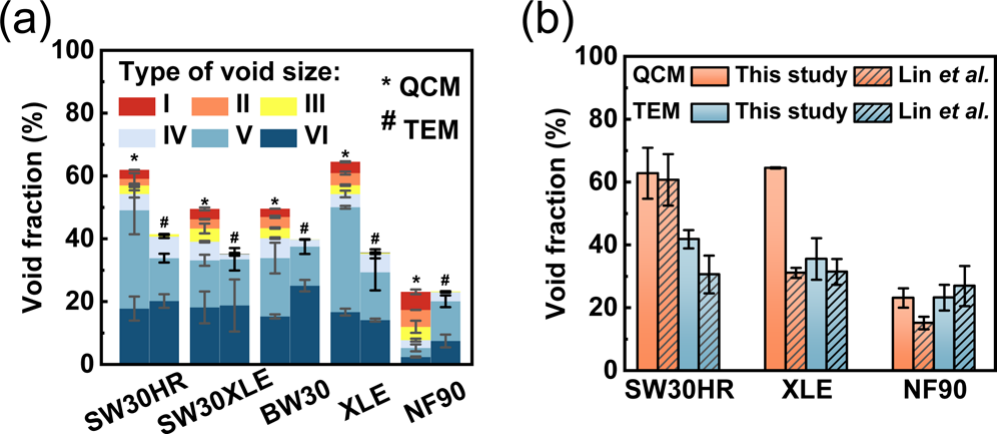
(a) Comparison of QCM-measured void fraction and TEM-measured void fraction of the commercial TFC membranes. (b) Comparison of void fractions for SW30HR, XLE, and NF90 membranes, as measured in this study and reported by Lin et al.^19^

The thickness of the polyamide films was also characterized using both TEM and QCM measurements for all the membranes (Figure 5). Traditionally, polyamide thickness is classified into intrinsic thickness (*t*_*int*_), i.e., the thickness of a single-layer polyamide film excluding voids, and apparent thickness (*t*_*app*_), i.e., the total height of the entire polyamide structure including voids (Figure 5a).^21^ In some studies, the term “ridge thickness” has also been used to describe the thickness of a collapsed polyamide leaf, which is approximately twice the intrinsic thickness.^27^ To further characterize the polyamide thickness, mass-based QCM measurements were employed to determine the thickness of the polyamide films (Figure 5a and Supporting Information S12). This thickness, termed as equivalent thickness (*t*_*eq*_), can be obtained by dividing the areal mass by the density of polyamide material. In the current study, a density of 1.3 g cm^–3^ is assumed.^40,45,46^ According to the experimental measurements by Zhou et al.,^40^ the density value is only weakly dependent on the cross-linking degree, showing an slight increase from 1.24 g cm^–3^ to 1.36 g cm^–3^ for polyamide films with elemental O/N ratios decreasing from 1.4 to 1.1. In the current study, the measured *t*_*eq*_ followed the order: SW30HR (165.4 ± 16.4 nm) > XLE (134.9 ± 13.6 nm) > BW30 (129.2 ± 29.1 nm) > NF90 (117.8 ± 2.9 nm) > SW30XLE (101.7 ± 3.3 nm), which was generally lower than *t*_*app*_ (Figure 5b). The QCM study by Lin et al.^47^ reported comparable thickness values, which are also lower than the respective values obtained from their TEM characterization. The difference in *t*_*app*_ and *t*_*eq*_ can be attributed to the contribution of nanovoid that is included in *t*_*app*_ but excluded in *t*_*eq*_. Besides, *t*_*eq*_ is obviously higher than *t*_*int*_ since the latter one considers only a single layer of the polyamide. Consequently, the QCM-derived *t*_*eq*_ generally falls between the TEM-based *t*_*int*_ and *t*_*app*_ (Figure 5b), with exception of SW30HR and BW30 that contain a surface coating of PVA.^11^

**Figure 5:**
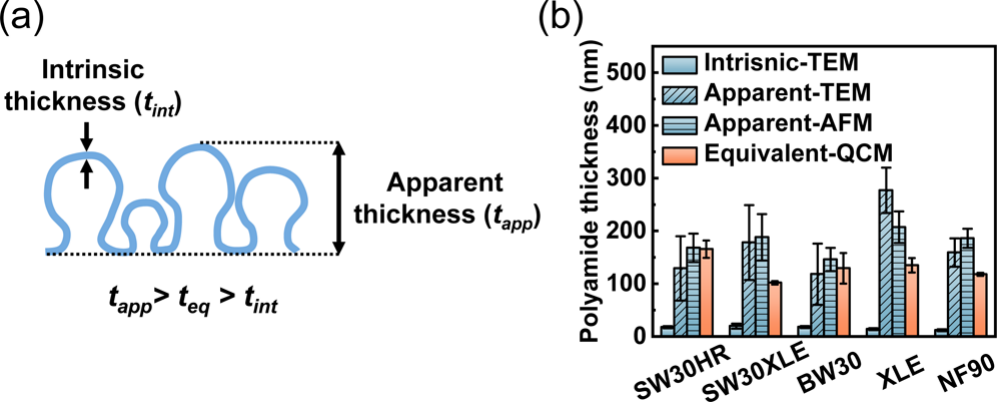
(a) Illustration of the intrinsic thickness (*t*_*int*_) and apparent thickness (*t*_*app*_) as determined from TEM image analysis. (b) Comparison of the TEM-measured intrinsic thickness, TEM-measured apparent thickness, and AFM-measured apparent thickness with the QCM-measured equivalent thickness of the commercial TFC membranes. The TEM-measured thickness for SW30XLE was obtained in the current study, while the values for the other membranes were collected from the literature.^21^ The AFM-measured apparent thickness represents the height difference between silicon wafer and polyamide layer (Figure S6).^48^ The equivalent thickness was calculated by dividing the areal mass density by the density of the polyamide film. A density of 1.3 g cm^–3^ was used in all calculations (see details in Supporting Information S12). Duplicate QCM tests were performed for each membrane type.

## Implications and Perspectives

The current study provides a novel approach for characterizing polyamide microstructures, including the quantification of nanovoids and their size distribution, which offers great potential for better understanding the intricate nanofoamed structure of TFC RO membranes. Conventional 2D TEM sectioning techniques often underestimate void size in polyamide films and fail to capture the full complexity of the 3D internal microstructure (Figure 6). Additionally, TEM imaging parameters, particularly the contrast between polyamide and background,^49^ could critically influence void quantification during image analysis. Variability in void fraction interpretation, stemming from differences in methods or individual biases (e.g., TEM sample preparation and cutting position, Figure S1 and Figure 6), can further lead to subjective and inaccurate results. These limitations underscore the need for advanced characterization techniques to achieve precise membrane void fraction assessments.

**Figure 6:**
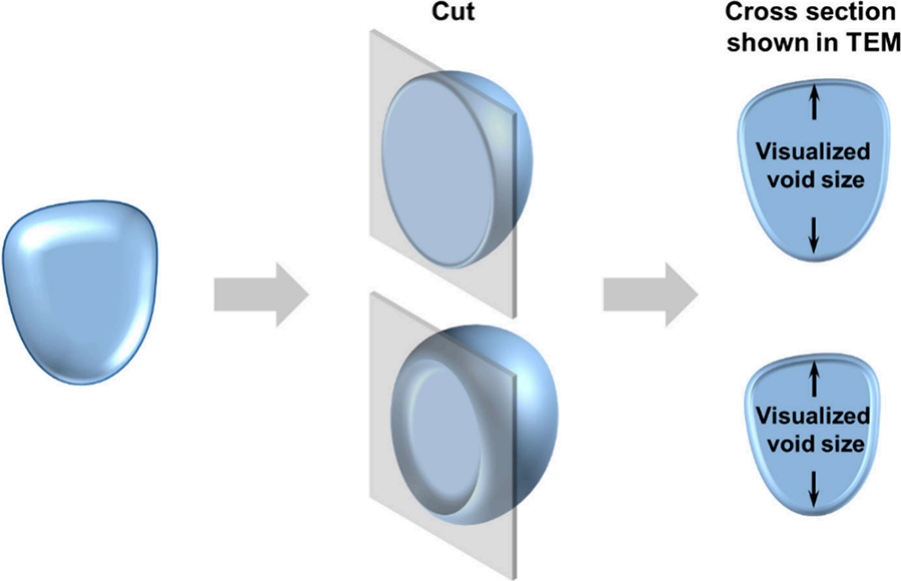
Illustration of the conventional 2D TEM sectioning techniques to quantify polyamide void sizes.^52^ The thickness of the TEM slice (∼100 nm) does not significantly affect void edge thickness, thus enabling relatively reliable void fraction measurements. However, void sizes are likely underestimated when cross sections are cut away from the void center.

Emerging 3D tomography methods (e.g., ET and cryo-ET),^22,23,27^ hold promise for revealing hidden empty spaces and defects within polyamide films that 2D analyses tend to overlook, providing a more comprehensive view of internal microstructures. However, ET is often used to present fully closed voids in the polyamide layer (Table S3). While cryo-ET can capture both open and enclosed voids, the challenges like high costs and complex sample preparation necessitate careful evaluation of their practicality for research applications. In contrast, QCM-based techniques offer distinct advantages, enabling simple and cost-effective quantification of void size distributions and total void fractions in polyamide films (Table 1). This method could complement 3D tomography to provide deeper insights into the membrane microstructures, which in turn facilitates better understanding of structure-performance relationship. Although it is beyond the scope of the current study, the QCM-based technique can be potentially extended to differentiate open vs closed voids by analyzing the rate of water condensation and evaporation, noting that close voids have much higher transport resistance^18^ and thus slower evaporation rate. Coupled with additional model development and experimental calibration, future studies may further develop this method for a more comprehensive analysis of polyamide structures. At the same time, the method may also be extended to other membrane types, such as gas separation^50^ and pervaporation.^51^

**Table 1:** Performance Comparison of 2D TEM, 3D TEM, and QCM-Based Methods in Terms of Cost Efficiency, Void Fraction, Void Size, and Thickness

			QCM	
Parameter	2D TEM	3D TEM^b^	Flow cell	Humidity cell
**Cost** ^a^	☆☆☆	☆	☆☆☆	☆☆
**Void** **fraction** ^a^	☆☆	☆☆☆	☆☆☆	☆☆☆
**Void** **size** ^a^	☆	☆☆☆	χ^c^	☆☆☆
**Thickness** ^a^	☆☆☆	☆☆☆	☆	☆

a The number of stars indicates the cost effectiveness or measurement accuracy of a method (☆, less effective; ☆☆, moderately effective; ☆☆☆, highly effective).

b 3D TEM characterization includes electron tomography (ET),^22^ machine learning-coupled ET,^23^ and cryo-ET.^27^

c QCM equipped with a flow cell is not applicable for measuring void size distribution.

## Supplementary Material



## Notes

Influence of cutting position on void size; mechanism of quartz crystal microbalance (QCM) humidity cell for nanovoid characterization; preparation of saturated salt solutions; isolation of polyamide selective layer and transfer on top of QCM gold sensor; void volume, void size distribution, and void fraction calculation; void fraction and void size distribution measurement from TEM images; mass change of the five commercial TFC membranes in the presence of different saturated salt solutions measured using the QCM method; separation performance, physicochemical properties, and QCM results of commercial polyamide TFC membranes; influence of dual-layer polyamide structure on TEM characterization; influence of active layer orientation on the characterization results of QCM method; comparison of the void fraction measured by different methods; and QCM measured equivalent thickness of the polyamide layers (PDF)
