# Supplementary material for: Quantification of Nanovoids in Fully-Aromatic Thin-Film Composite Polyamide Membranes Using a Quartz Crystal Microbalance Equipped with a Humidity Cell
Source: Environ Sci Technol. 2026 Jul 25;60(31):22000–8. doi: 10.1021/acs.est.6c00927 (PMC13472174; doi:10.1021/acs.est.6c00927)
Supplement: Supplementary file 1 [file es6c00927_si_001.pdf]

SUPPORTING INFORMATION

Quantification of Nanovoids in Fully-Aromatic Thin-film Composite Polyamide Membranes using Quartz Crystal Microbalance Equipped with Humidity Cell

Chenyue Wu,[a1] Zhe Yang,[a,b1] Lu Elfa Peng,[a] Pulak Sarkar,[a,c]* and Chuyang Y. Tang[a,d]*

[a] Department of Civil Engineering, The University of Hong Kong, Pokfulam, Hong Kong SAR, China

[b] Department of Civil and Environmental Engineering, The Hong Kong University of Science and Technology, Hong Kong SAR, China

[c] Department of Chemical and Biological Engineering, The Hong Kong University of Science and Technology, Hong Kong SAR, China

[d] Department of Civil & Environmental Engineering, National University of Singapore, Singapore 117576

[1] Chenyue Wu and Zhe Yang contributed equally.

* Corresponding Author

Phone: +852 2859 1976, E-mail address: cy_tang@nus.edu.sg

Phone: +852 5619 9591, E-mail address: pulaks@ust.hk

Number of pages: 20

Number of figures: 8

Number of tables: 4

Contents:

S1. Influence of cutting position on void size (Page S3)
S2. Mechanism of quartz crystal microbalance (QCM) humidity cell for nanovoid characterization (Page S4)
S3. Preparation of saturated salt solutions (Page S6)
S4. Isolation of polyamide selective layer and transfer on top of QCM gold sensor (Page S7)
S5. Void volume, void size distribution, and void fraction calculation (Page S8)
S6. Void fraction and void size distribution measurement from TEM images (Page S9)
S7. Mass change of the five commercial TFC membranes in the presence of different saturated salt solutions measured using the QCM method (Page S10)
S8. Separation performance, physicochemical properties, and QCM results of commercial polyamide TFC membranes (Page S11)
S9. Influence of dual-layer polyamide structure on TEM characterization (Page S14)
S10. Influence of active layer orientation on the characterization results of QCM method (Page S15)
S11. Comparison of the void fraction measured by different methods (Page S17)
S12. QCM measured equivalent thickness of the polyamide layers (Page S18)
S13. References (Page S19)

S1. Influence of cutting position on void size:

In practice, it is unlikely for a thin TEM section to cut through the centers of all voids simultaneously. For a section that cuts away from the center of a void, the corresponding void size tends to be underestimated (Figure S1).

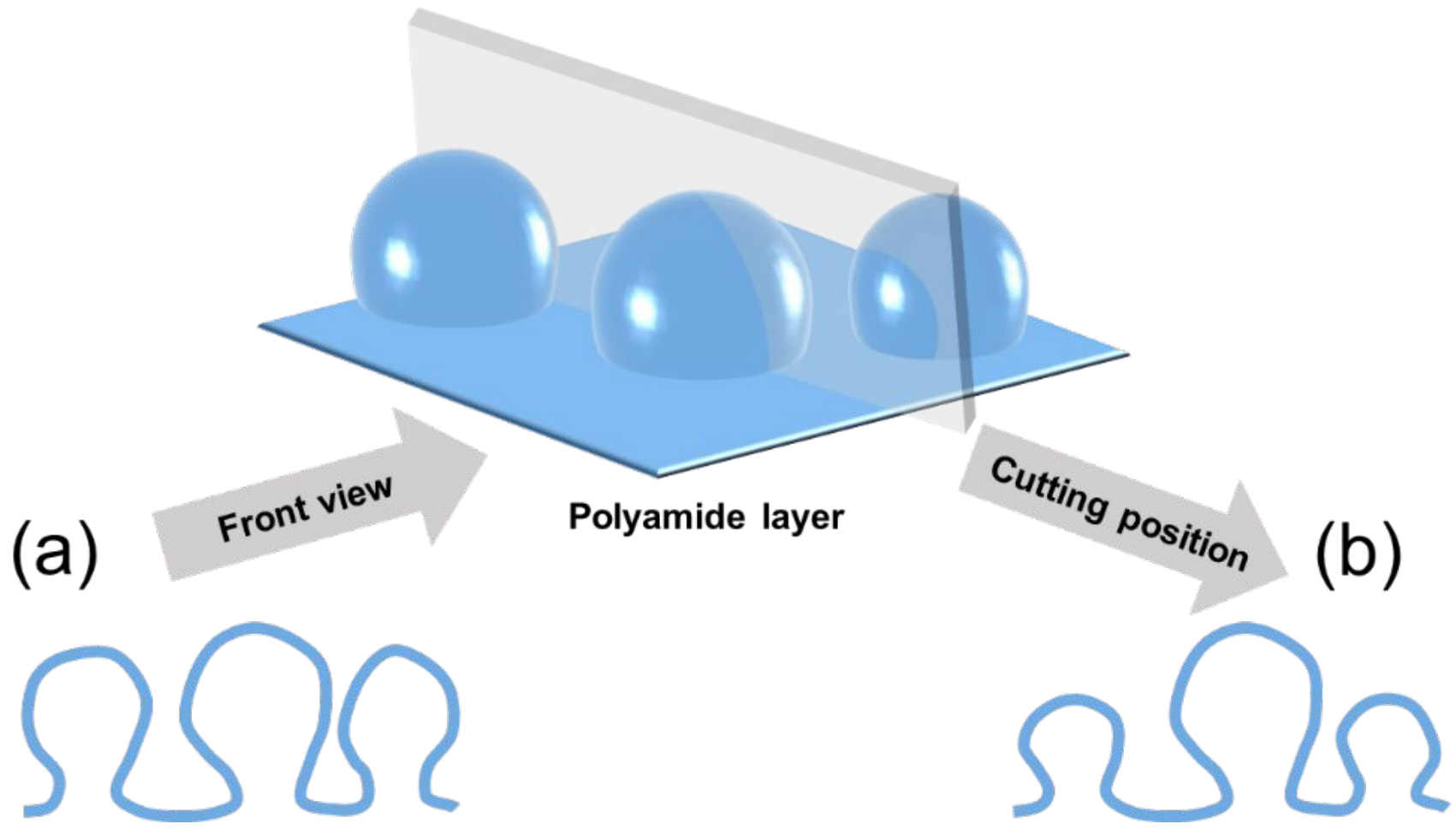

Figure S1. (a) Front-view schematic of cross-sectional void sizes in the polyamide layer, where all voids are represented by great circle cross-sections (cut through the center of the void). (b) Void sizes observed at the cutting position of the TEM sample slice, including both great and small circles.

S2. Mechanism of quartz crystal microbalance (QCM) humidity cell for nanovoid characterization:

Q-Sense's Humidity Module is a specialized measurement chamber designed for rapid, accurate, and non-destructive analysis of water vapor uptake and release in thin films, enabling detailed characterization of their nanoscale porosity (Figure S2a,b). The humidity cell consists of a dual-chamber system separated by a highly permeable hydrophobic GORE^{TM} membrane (W.L. Gore & Associates, Newark, DE) that allows controlled water vapor exchange while preventing liquid cross-over. This configuration creates an air-filled vapor compartment above the polyamide film-coated sensor (Figure S2c). During experiments, the polyamide layer is indirectly exposed to aqueous solutions through the vapor phase, ensuring that interactions occur via water vapor diffusion across the GORE^{TM} membrane.

When a saturated salt solution is injected into the liquid compartment, water vapor in the vapor compartment interacts with the polyamide layer, leading to dynamic mass changes driven by water condensation and evaporation within the polyamide matrix and its nanovoids. For example, injecting deionized (DI) water with 100% relative humidity (RH) results in complete saturation of the polyamide layer and its nanovoids with water. Conversely, when the saturated film is exposed to a lower RH environment, water evaporates from the nanovoids, reducing the mass until the system reaches thermodynamic equilibrium between the liquid and vapor compartments. These mass changes are detected by the QCM, which measures frequency shifts proportional to nanogram scale mass changes. By systematically decreasing RH, water is selectively evaporated from nanovoids of specific sizes, as smaller voids retain water at lower RH due to stronger capillary condensation.[1] According to the Kelvin equation, the equilibrium water vapor pressure and thus RH are directly correlated with pore size or nanovoid size. Monitoring the real-time mass changes allows for their conversion into volume, providing a quantitative assessment of void size and distribution within the polyamide film.

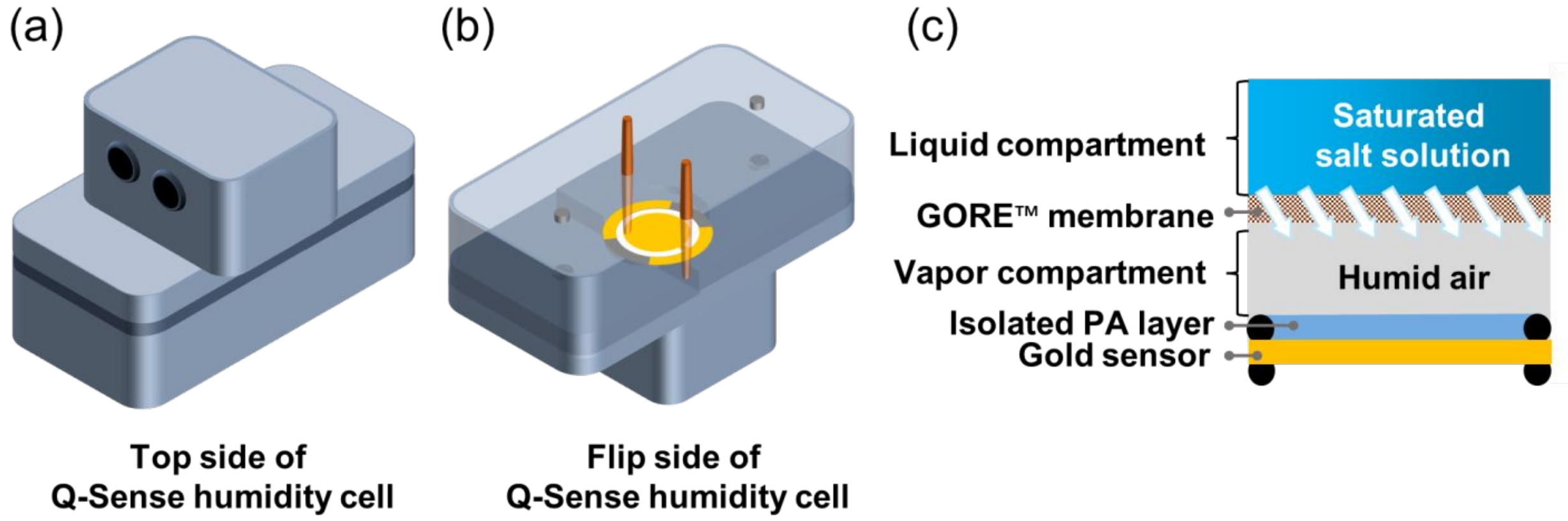

Figure S2. (a,b) Schematic illustration of the Q-Sense humidity cells used in this study. (c) Schematic illustration of the humidity cell showing the GORE^{TM} membrane that separates the sensor from the liquid flow.

S3. Preparation of saturated salt solutions:

Potassium sulfate (K_2SO_4), potassium nitrate (KNO_3), potassium chloride (KCl), sodium chloride (NaCl), magnesium chloride ($MgCl_2$), and lithium chloride (LiCl) were used to prepare saturated solutions for humidity control in the QCM test. All aqueous solutions were prepared using deionized (DI) water produced from the Milli-Q system with a resistivity of 18.2 MΩ cm (Millipore, US). Detailed information regarding the chemicals and saturated salt solutions is provided in Table S1.

Table S1. Details of the provider, purity, solubility of the six salts, temperature of prepared saturated salt solutions, and the resulting relative humidity (RH) in the QCM humidity cell.

Chemical	Provider	Purity	Solubility in water (g/100 ml)	Temperature (°C)	RH (%)
K_2SO_4	Dieckmann, China	AR*	12	25	97.3
KNO_3	VWR, US	AR*	36	25	93.6
KCl	Dieckmann, China	AR*	34	25	84.3
NaCl	Dieckmann, China	≥ 99.8%	36	25	75.3
$MgCl_2$	UniChem, New Zealand	AR*	54	25	32.8
LiCl	TCI, US	98%	84	25	11.3

* Analytical reagent.

S4. Isolation of Polyamide Selective Layer and Transfer on Top of QCM Gold Sensor:

To characterize the polyamide selective layer by QCM (Q-Sense, Biolin Scientific, Sweden), DMF was used to dissolve the substrate and isolate the selective layer from the composite membrane. Figure S3 illustrates the isolation process of the polyamide selective layer from the composite structure.[2, 3] Briefly, the non-woven fabric was first peeled off from the composite structure. A 1.0 cm^2 section of the polyamide film, along with its porous back layer (e.g., polysulfone), was then placed onto the QCM sensor, ensuring the polyamide surface faced the sensor. After installing the sensor onto a custom-made stainless steel setup,[4] DMF was applied for 3 min to dissolve the porous back layer.[2, 5] The DMF rinsing process was repeated at least 20 times until no residue was visible.

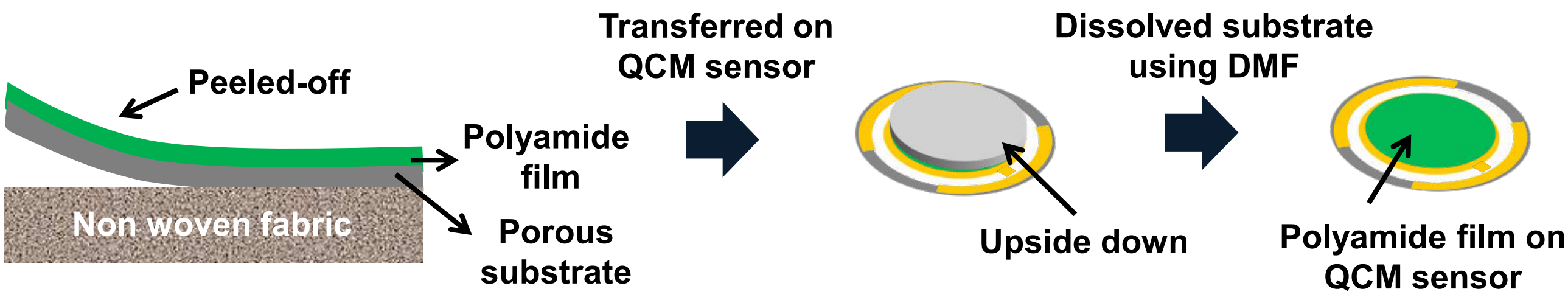

Figure S3. Schematic illustration of the polyamide layer isolation and transfer process onto the QCM gold sensor.

S5. Void volume, void size distribution, and void fraction calculation:

The void volume associated with a specific RH that corresponds to a specific void radius was calculated from the mass change. The volume of adsorbed water (V_w) can be calculated as:

$$V_w = \frac{\Delta m}{\rho_w} \quad \text{(S1)}$$

Assuming the adsorbed water completely fills the voids, the volume of water (V_w) equals to the void volume (V_p):

$$V_p = V_w = \frac{\Delta m}{\rho_w} \quad \text{(S2)}$$

where ρ_w is the density of water (1.0 g cm^{-3}).

The total void volume was calculated by adding up the individual void volumes measured across the entire range of RH conditions tested, i.e., from a fully saturated state (e.g., exposed to DI water) to a low RH condition (e.g., using a LiCl solution). The percentage of mass shifts related to voids available at a given RH is determined by the ratio of the mass shift at that RH to the total mass shift observed over the entire humidity range, offering insight into the distribution of void sizes.

Additionally, the void fraction (f_{void}) within the polyamide layer was calculated based on water uptake under DI water exposure. The following equation was used to quantify the percentage of the membrane volume occupied by void, offering a complementary measure of its void fraction.

$$f_{void} = \frac{void\ volume}{Polyamide\ volume} = \frac{m_w/\rho_w}{m_w/\rho_w + m_{polyamide}/\rho_{polyamide}} \quad \text{(S3)}$$

where m_w (ng cm^{-2}) and $m_{polyamide}$ (ng cm^{-2}) correspond to the water mass uptake when polyamide layers were exposed to liquid water and the dry mass of the polyamide selective layer. The density of water (ρ_w) and polyamide layer ($\rho_{polyamide}$) is 1.0 g cm^{-3} and 1.3 g cm^{-3}, respectively.[6]

S6. Void fraction and void size distribution measurement from TEM images:

Figure S4a illustrates an example of a grey-scale transmission electron microscopy (TEM) image of a polyamide TFC membrane (Figure S4a). To distinguish the polyamide region, the brightness values of the TEM image were analyzed. Using Image-Pro Plus software (Media Cybernetics, Inc.), the darker areas (e.g., brightness value < 90) were highlighted in red (Figure S4b). The remaining regions, representing the nanovoids within the polyamide layer, were manually highlighted in yellow (Figure S4c). Image-Pro Plus software was then used to determine the area of the polyamide region and voids. The following equation was used to determine the void fraction (ε, %):

$$\varepsilon = \frac{\sum A_{voids}}{A_{polyamide}} \cdot 100\% \qquad \text{(S4)}$$

where A_{voids} is the area of each void, and $A_{polyamide}$ is the area of the polyamide layer (including the void area). Essentially, the Image-Pro Plus software, used for image analysis in the current study, reports the average diameter of a circle in several directions if the subject is not perfectly circular in shape. To ensure reliability, the void size and void fraction were obtained based on images from two independent TEM slices.

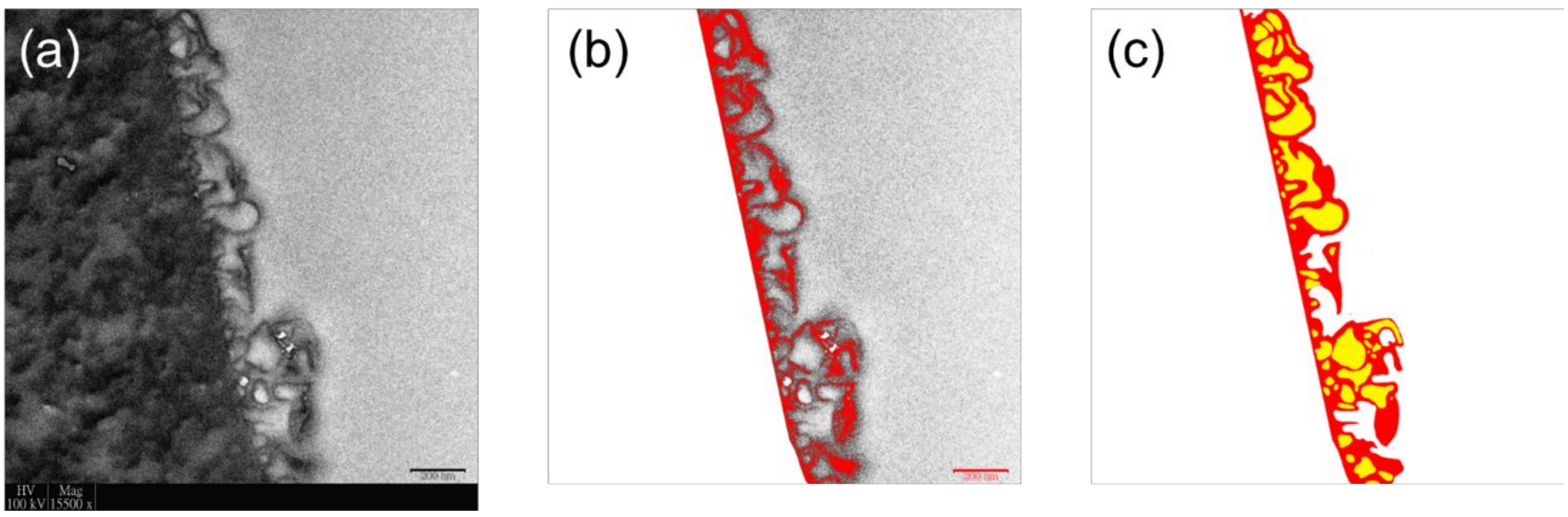

Figure S4. (a) TEM image of the BW30 membrane. Measurement of (b) polyamide area and (c) voids area based on TEM images of the BW30 membrane using Image-Pro Plus software.

S7. Mass change of the five commercial TFC membranes in the presence of different saturated salt solutions measured using the QCM method:

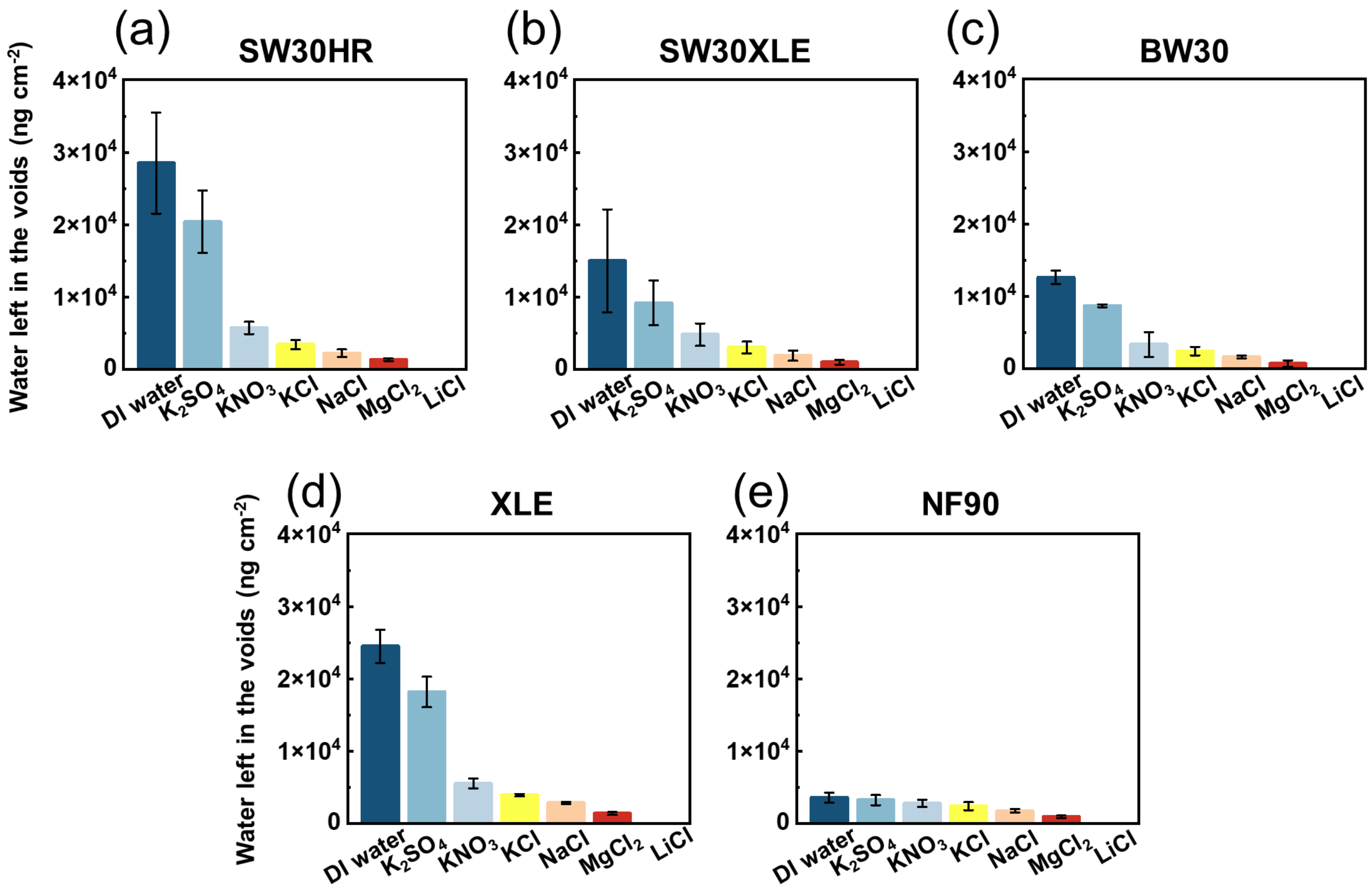

Figure S5. The mass of water left in the voids of (a) SW30HR, (b) SW30XLE, (c) BW30, (d) XLE, and (e) NF90 membranes during water desorption process, which is measured by QCM after injecting different solutions such as DI water (RH = 100%), K_2SO_4 (RH = 97.3 %), KNO_3 (RH = 93.6 %), KCl (RH = 84.3%), NaCl (RH = 75.3%), $MgCl_2$ (RH = 32.8%), and LiCl (RH = 11.3%) in the liquid compartment.

S8. Separation performance, physicochemical properties, and QCM results of commercial polyamide TFC membranes:

The separation performance of the polyamide TFC membranes, including both pure water permeance and salt rejection (NaCl), was tested in a cross-flow filtration setup.[7] Each membrane coupon with an effective filtration area of 12.0 cm^2 was pre-compacted at 17.5 bar pressure for 2 hours. Filtration tests were then conducted at 15.5 bar using DI water, maintaining a cross-flow velocity of approximately 22.4 cm s^{-1} and a feed temperature of 25 (± 1) °C. The pure water flux (J_w, L m^{-2} h^{-1}) of the membrane was calculated using Eq. (S5):

$$J_w = \frac{\Delta V}{\Delta t \times S} \quad \text{(S5)}$$

where V (L) is the volume of permeate, Δt (h) is the filtration time, and S (m^2) is the effective filtration area. The pure water permeance (A, L m^{-2} h^{-1} bar^{-1}) was calculated by the following equation:

$$A = \frac{J_w}{\Delta P} \quad \text{(S6)}$$

where ΔP (bar) is the applied hydraulic pressure.

To assess the salt rejection performance of the commercial membranes, a feed solution of 2000 ppm NaCl was used. The rejection (R, %) of the membranes was calculated based on the concentration difference between the feed and permeate solutions using Eq. (S7):

$$R = \frac{C_f - C_p}{C_f} \times 100\% \quad \text{(S7)}$$

where C_p and C_f are the salt concentrations in the permeate and feed solution. The salt concentration was determined by measuring conductivity (µS cm^{-1}) with a conductivity meter (Ultrameter II, Myron L).

Table S2. Separation performance, physicochemical properties, and QCM results of five commercial polyamide TFC membranes.

Properties			Membranes				
			SW30HR	SW30XLE	BW30	XLE	NF90
Performance	WP ($L\ m^{-2}\ h^{-1}\ bar^{-1}$)		0.7 ± 0.2	1.1 ± 0.2	3.5 ± 0.6	6.3 ± 0.3	6.9 ± 1.1
	NaCl rejection (%)		98.5 ± 0.2	98.3 ± 0.6	97.2 ± 0.5	93.1 ± 0.9	82.8 ± 1.7
	O/N		3.2 ± 0.1	3.9 ± 0.1	3.8 ± 1.1	1.6 ± 0.1	1.6 ± 0.1
	CA (°)		33.1 ± 1.0	45.6 ± 3.6	42.8 ± 3.6	100.2 ± 8.1	79.5 ± 2.0
TEM	t_{app} (nm)*		129.0 ± 61.0	178.0 ± 71.0	118.0 ± 58.0	277.0 ± 43.0	159.0 ± 27.0
	t_{int} (nm)*		17.4 ± 2.0	19.6 ± 4.8	17.8 ± 1.9	13.9 ± 1.8	11.8 ± 1.7
	Void volume ($nL\ cm^{-2}$)	Type I	0	0	0	0	0
		Type II	0	0	0	0	0
		Type III	0.1 ± 0.0	0	0	0.1 ± 0.1	0
		Type IV	0.9 ± 0.1	0.2 ± 0.1	0.5 ± 0.1	1.5 ± 0.2	0.7 ± 0.1
		Type V	1.8 ± 0.1	2.1 ± 0.8	2.6 ± 0.8	3.8 ± 0.9	2.8 ± 0.7
		Type VI	2.7 ± 0.1	2.4 ± 0.8	5.2 ± 1.1	3.7 ± 0.7	1.7 ± 0.6
	Total void volume ($nL\ cm^{-2}$)		5.5 ± 0.1	4.7 ± 0.1	8.3 ± 2.0	9.1 ± 0.3	5.2 ± 1.4
	Void fraction (%)		41.5 ± 2.9	35.1 ± 4.2	39.8 ± 4.2	35.6 ± 6.6	23.3 ± 4.1
AFM	R_a (nm)		68.8 ± 15.0	71.0 ± 5.8	48.9 ± 7.2	64.4 ± 13.0	52.2 ± 15.4
	t_{app} (nm)		168.8 ± 27.7	188.8 ± 45.0	146.0 ± 22.9	207.7 ± 30.6	186.7 ± 18.2
QCM	Mass ($\mu g\ cm^{-2}$)		21.5 ± 2.1	13.2 ± 0.4	16.8 ± 3.8	17.5 ± 1.8	15.3 ± 0.4
	t_{eq} (nm)		165.4 ± 16.4	101.7 ± 3.3	129.2 ± 29.1	134.9 ± 13.6	117.8 ± 2.9
	Void volume ($nL\ cm^{-2}$)	Type I	1.28 ± 0.17	0.66 ± 0.10	0.66 ± 0.1	1.35 ± 0.20	0.88 ±0.17
		Type II	0.90 ± 0.70	0.60 ± 0.03	0.92 ± 0.92	1.47 ± 0.05	0.83 ± 0.06
		Type III	1.21 ± 0.10	0.85 ± 0.33	0.81 ± 0.07	1.06 ± 0.01	0.65 ± 0.34
		Type IV	2.3 ± 0.24	1.19 ± 0.07	1.62 ± 0.31	1.61 ± 0.55	0.38 ± 0.05
		Type V	14.68 ± 5.17	3.02 ± 0.40	4.65 ± 0.53	12.68 ± 1.38	0.45 ± 0.20
		Type VI	8.13 ± 2.68	3.65 ± 0.97	3.91 ± 0.76	6.31 ± 0.19	0.36 ± 0.01
	Total void volume ($nL\ cm^{-2}$)		28.5 ± 7.0	15.0 ± 7.1	12.6 ± 0.9	24.5 ± 2.3	3.6 ± 0.7
	Void fraction (%)		62.8 ± 8.1	49.5 ± 0.9	49.5± 3.8	64.5 ± 0.2	23.1 ± 3.1

Note: WP = Water permeance; t_{app} = Apparent thickness; t_{int} = Intrinsic thickness; t_{eq} = equivalent thickness (the calculation method is provided in Supporting Information S12). Six void size categories were defined based on their ranges: Type I (<1.7 nm), Type II (1.7 – 6.7 nm), Type III (6.7 – 11.2 nm), Type IV (11.2 – 28.7 nm), Type V (28.7 – 69.6 nm), and Type VI (> 69.6 nm). The AFM-measured apparent thickness represents the height difference between silicon wafer and polyamide layer,[8] which

is shown in Figure S6. The thickness denoted with an asterisk (*) for SW30XLE were measured in the current study, while the values for the other membranes were collected from the literature.[9]

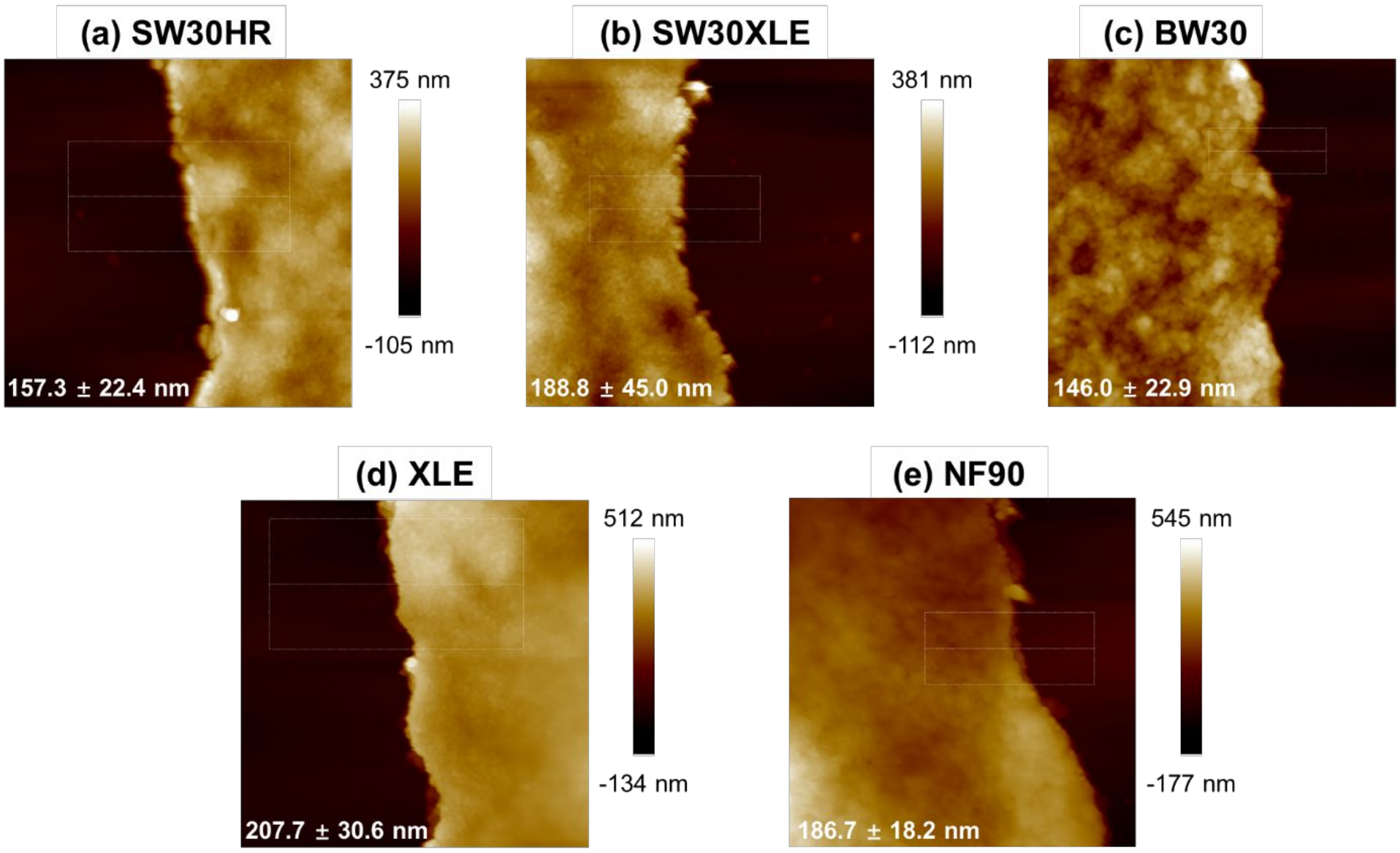

Figure S6. Atomic force microscopy (AFM) images of polyamide films isolated on silicon wafers. The membrane substrate was dissolved using DMF as described in Supporting Information S4. To determine the polyamide thickness by measuring the distance between the silicon wafer and the polyamide layer, all films were sectioned by scratching the surface with a metal needle.[8]

S9. Influence of dual-layer polyamide structure on TEM characterization:

The preparation of TEM slices may lead to misinterpretation of nanovoid sizes. A typical TEM slice thickness is approximately 100 nm. Therefore, it may include several adjacent leaf-like features from the exterior layer, which can complicate the interpretation of void identification (Figure S7). For example, for the NF90 membrane, the boundary of small nodules in the lower (basal) layer can be more easily identified. In contrast, it can be difficult to resolve the leaf-like features in the exterior layer. As illustrated in Figure S7, when two adjacent leaf-like features are projected into a 2D image, they could be mistakenly taken as a single larger feature. As a result, the open space "underneath" the exterior layer may sometimes be falsely identified as void volume, resulting in an overestimation of larger voids (particularly Types V and VI).

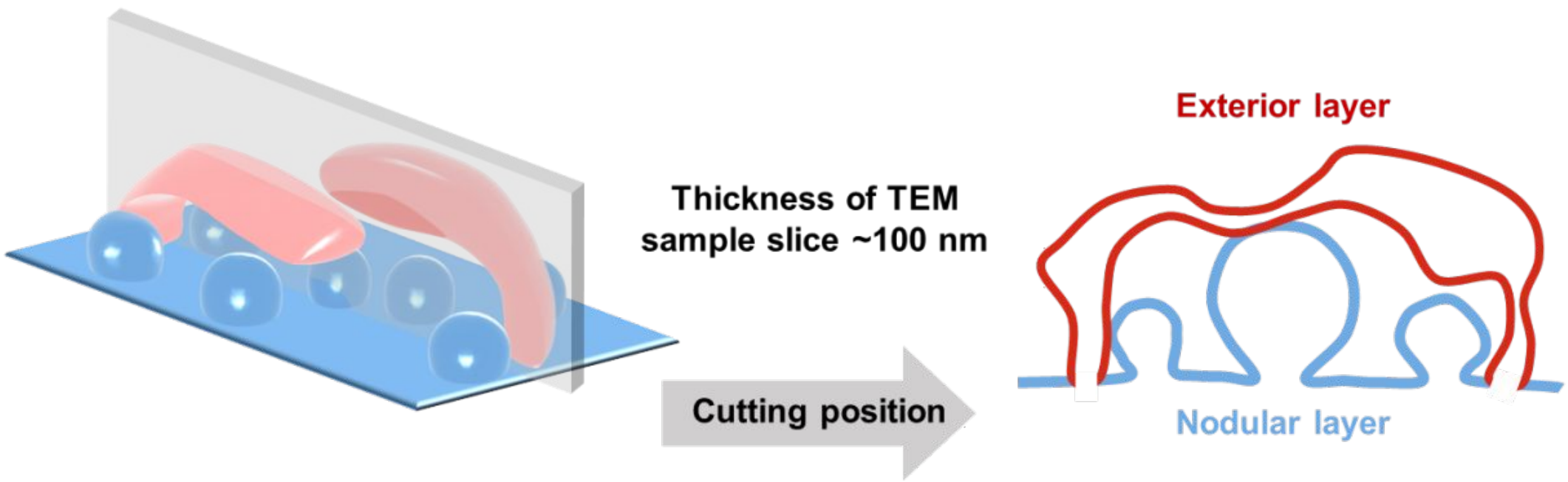

Figure S7. Schematic diagram illustrates the influence of cutting position on the TEM image of a dual-layer polyamide. The lower nodular layer is highlighted in blue, and the upper exterior layer is highlighted in red.

S10. Influence of active layer orientation on the characterization results of QCM method:

To investigate the influence of active layer orientation in QCM measurements,[10] we performed an additional test for the alternative orientation. A support-free method was used to load the top polyamide nanofilm of a commercial membrane onto a gold sensor (active side facing up).[11] First, the nonwoven backing of the commercial membrane was peeled off. The resulting bilayer (polysulfone and polyamide) was placed with the polyamide side contacting a smooth polytetrafluoroethylene (PTFE) support membrane and then clamped between a glass base and a glass funnel. Subsequently, the polysulfone support layer was dissolved using DMF. This dissolution step was repeated ten times, each involving immersion in 10 mL of DMF for 2 minutes, to ensure complete removal of polysulfone.

Before transferring the polyamide membrane onto the sensor, a free water/hexane interface was established in a custom filtration device with a microfiltration membrane underneath. A gold wafer placed on a glass slide was positioned at the center of the microfiltration membrane. The PTFE-supported polyamide nanofilm was then quickly inverted and carefully transferred onto the immiscible liquid interface. The PTFE support membrane was gently removed with tweezers, and the polyamide nanofilm was deposited onto the gold wafer by vacuum filtration of water. The hexane layer was subsequently removed using a pipette. Finally, the polyamide-coated sensor was dried in room temperature until completely dry.

Figure S8 presents the QCM-measured void size distributions of the NF90 membrane with the active layer facing down or up. Both orientations show a similar trend of greater presence of smaller voids (Types I–III) compared to larger voids (Types IV–VI). Moreover, the void volumes for each type and the total void fraction are comparable between the two orientations. These results indicate that the active layer orientation has no significant effect on nanovoid characterization.

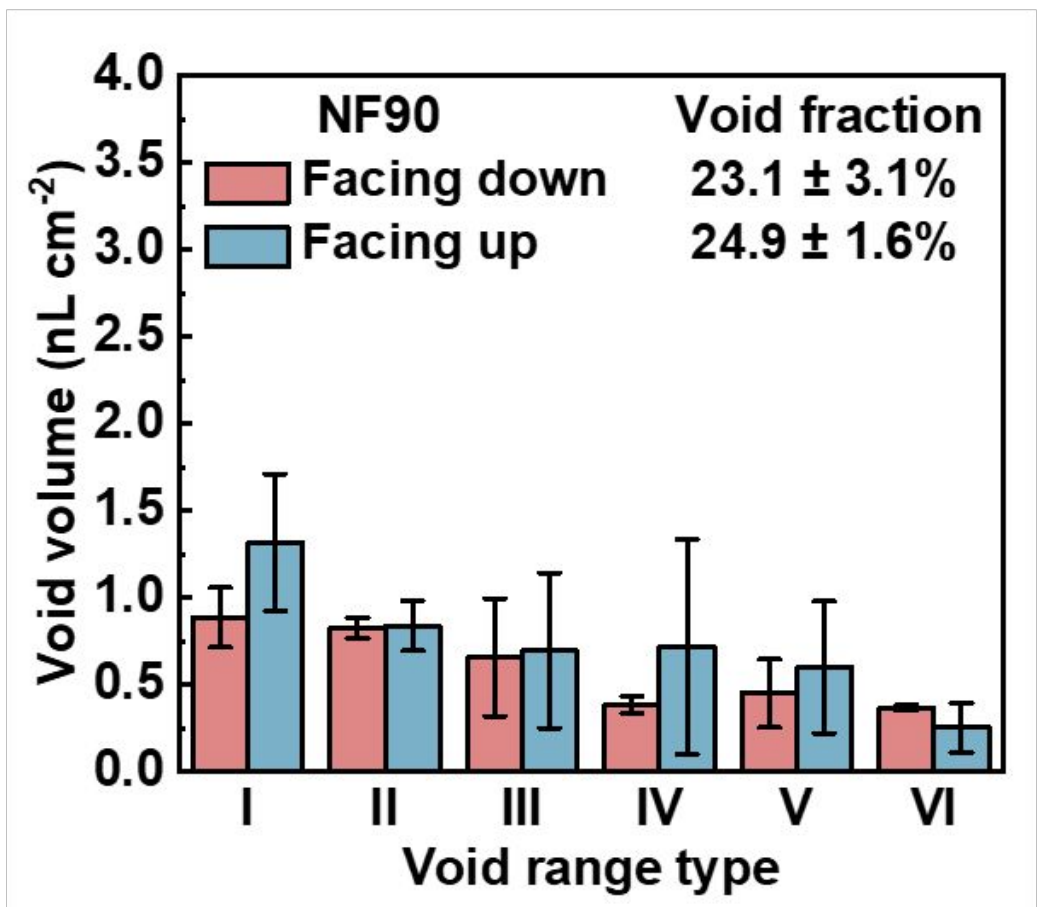

Figure S8. Comparison of the QCM-measured void size distribution of the NF90 polyamide layer with the active layer facing down (i.e., facing the gold sensor) versus facing up (i.e., facing the vapor compartment).

S11. Comparison of the void fraction measured by different methods:

Table S3. The void fractions of commercial polyamide TFC membranes measured using different methods (e.g., electron tomography or ET, TEM, and QCM) reported in the literature.

Method	Void fraction (%)				
	SW30HR	SW30XLE	BW30	XLE	NF90
Machine learning & ET[12]	N.A.	0.79[#]	0.54[#]	N.A.	N.A.
ET[13]	0.12[#]	N.A.	N.A.	0.04[#]	N.A.
Cryo-ET[14]	N.A.	22.1	18.7	N.A.	N.A.
TEM	41.5* (30.0 [ref. 15], 30.1 [ref. 6])	35.1*	39.8*	35.6* (32.0 [ref. 15], 31.7 [ref. 6])	23.3* (25.7 [ref. 6])
QCM	61.7* (60.3 [ref. 6])	48.3*	48.4*	63.4* (30.1 [ref. 6])	22.3* (15.4 [ref. 6])

[#] These values only represent the percentage of fully closed voids in the polyamide layer. In comparison, other reported values include both fully closed and semi-closed voids.

* The values outside the brackets were measured in this study, and those inside the brackets were obtained from the literature.

S12. QCM measured equivalent thickness of the polyamide layers:

The results of the equivalent thickness are shown in Table S4. The equivalent thickness measured by QCM is calculated by dividing the areal mass density by the density of the loaded polyamide layers using Eq. (S8):

$$t_{eq} = \frac{m}{A}/\rho_{polyamide} \quad \text{(S8)}$$

Table S4. Result of the equivalent thickness of the polyamide film measured using QCM mass-based experiment of the commercial TFC membranes.[2, 16]

Membrane	Frequency change, Δf (Hz)	Areal mass density of the polyamide film, $\frac{m}{A}$ (ng cm^{-2})	Density of the polyamide film, $\rho_{polyamide}$ (g cm^{-3})	Equivalent thickness, t_{eq} (nm)
SW30HR	1215 ± 120	21505 ± 2127	1.3	165.4 ± 16.4
SW30XLE	747 ± 24	13222 ± 425	1.3	101.7 ± 3.3
BW30	949 ± 213	16797 ± 3779	1.3	129.2 ± 29.1
XLE	991 ± 99.7	17532 ± 1765	1.3	134.9 ± 13.6
NF90	865 ± 21	15310 ± 375	1.3	117.8 ± 2.9

S13. References:

(1) Evans, R.; Marconi, U. M. B.; Tarazona, P. Capillary condensation and adsorption in cylindrical and slit-like pores. *Journal of the Chemical Society, Faraday Transactions 2: Molecular and Chemical Physics* **1986**, *82* (10), 1763-1787.

(2) Sarkar, P.; Modak, S.; Karan, S. Ultraselective and Highly Permeable Polyamide Nanofilms for Ionic and Molecular Nanofiltration. *Adv. Funct. Mater.* **2021**, *31* (3), 2007054.

(3) Freger, V. Swelling and Morphology of the Skin Layer of Polyamide Composite Membranes: An Atomic Force Microscopy Study. *Environ. Sci. Technol.* **2004**, *38* (11), 3168-3175.

(4) Long, L.; Wu, C.; Yang, Z.; Tang, C. Y. Carbon nanotube interlayer enhances water permeance and antifouling performance of nanofiltration membranes: mechanisms and experimental evidence. *Environ. Sci. Technol.* **2022**, *56* (4), 2656-2664.

(5) Lin, L.; Feng, C.; Lopez, R.; Coronell, O. Identifying facile and accurate methods to measure the thickness of the active layers of thin-film composite membranes – A comparison of seven characterization techniques. *J. Membr. Sci.* **2016**, *498*, 167-179.

(6) Lin, L.; Lopez, R.; Ramon, G. Z.; Coronell, O. Investigating the void structure of the polyamide active layers of thin-film composite membranes. *J. Membr. Sci.* **2016**, *497*, 365-376.

(7) Peng, L. E.; Jiang, Y.; Wen, L.; Guo, H.; Yang, Z.; Tang, C. Y. Does interfacial vaporization of organic solvent affect the structure and separation properties of polyamide RO membranes? *J. Membr. Sci.* **2021**, *625*, 119173.

(8) Ritt, C. L.; Werber, J. R.; Wang, M.; Yang, Z.; Zhao, Y.; Kulik, H. J.; Elimelech, M. Ionization behavior of nanoporous polyamide membranes. *Proc. Natl. Acad. Sci.* **2020**, *117* (48), 30191-30200.

(9) Song, X.; Gan, B.; Qi, S.; Guo, H.; Tang, C. Y.; Zhou, Y.; Gao, C. Intrinsic Nanoscale Structure of Thin Film Composite Polyamide Membranes: Connectivity, Defects, and Structure-Property Correlation. *Environ. Sci. Technol.* **2020**, *54* (6), 3559-3569.

(10) Villalobos, L. F.; Pataroque, K. E.; Pan, W.; Cao, T.; Kaneda, M.; Violet, C.; Ritt, C. L.; Hoek, E. M. V.; Elimelech, M. Orientation matters: Measuring the correct surface of polyamide membranes with quartz crystal microbalance. *J. Membr. Sci. Lett.* **2023**, *3* (2).

(11) Wang, L. A.; Yang, Z.; Guo, H.; Liu, W.; Xiao, Q.; Tang, C. Y. EQCM-D assisted characterization of polyamide nanofiltration membrane: Mechanistic insights of solutes transport under various pH conditions. *J. Membr. Sci.* **2026**, *741*.

(12) Li, D.; Lu, R.; Wang, K.; Li, Y.; Lin, W.; Wang, X.-m.; Shen, Y.-x.; Huang, X. Neural Network-Assisted Data Processing Improved Tomography Characterizations of Reverse Osmosis Polyamide Layers. *ACS ES&T Engg.* **2023**, *4* (1), 128-138.

(13) Culp, T. E.; Shen, Y.-x.; Geitner, M.; Paul, M.; Roy, A.; Behr, M. J.; Rosenberg, S.; Gu, J.; Kumar, M.; Gomez, E. D. Electron tomography reveals details of the internal microstructure of desalination membranes. *Proc. Natl. Acad. Sci.* **2018**, *115* (35), 8694-8699.

(14) Li, D.; Liu, W.; Wang, X.; Lin, W.; Zhai, J.; Fan, H.; Xiao, K.; Wang, K.; Li, Y.; Jin, Y.; et al. Nodular networks in hydrated polyamide desalination membranes enhance water transport. *Sci. Adv.* **2025**, *11* (18), eadt3324.

(15) Wong, M. C. Y.; Lin, L.; Coronell, O.; Hoek, E. M. V.; Ramon, G. Z. Impact of liquid-filled voids within the active layer on transport through thin-film composite membranes. *J. Membr. Sci.* **2016**, *500*, 124-135.

(16) Zhou, S.; Long, L.; Yang, Z.; So, S. L.; Gan, B.; Guo, H.; Feng, S.-P.; Tang, C. Y. Unveiling the growth of polyamide nanofilms at water/organic free interfaces: toward enhanced water/salt selectivity. *Environ. Sci. Technol.* **2022**, *56* (14), 10279-10288.
